# Flexural strength and fracture load of high-flow bulk-fill and conventional composites in ISO 4049 bars and MOD cavity models: an in vitro study

**DOI:** 10.1186/s12903-026-08494-1

**Published:** 2026-04-30

**Authors:** Burcu Öztürk, Elif Aslıhan Kahraman Karşıgil, Fulya Aydın

**Affiliations:** 1 Department of Restorative Dentistry, Faculty of Dentistry, Ankara University, Ankara, Türkiye; 2 Department of Restorative Dentistry, Faculty of Dentistry, Ankara Medipol University, Ankara, Türkiye

**Keywords:** High-flow bulk-fill composite, Flexural strength, Fracture load, ISO 4049, MOD cavity model

## Abstract

**Background:**

High-flow bulk-fill (HFB) resin composites have been introduced to simplify restorative procedures by enabling bulk placement and improved cavity adaptation. However, evidence is limited regarding whether their mechanical performance as stand-alone materials is consistent across different testing geometries. This study aimed to compare the flexural strength and fracture load of HFB composites with a conventional paste-like posterior composite using ISO 4049 bar specimens and MOD shaped restorations.

**Methods:**

Eighty specimens were fabricated and divided into two specimen configurations: ISO 4049 bar specimens (25 × 2 × 2 mm) and MOD shaped restorations (12 × 7 × 5 mm) (*N* = 40 each). Three HFB composites—Clearfil Majesty ES High Flow (CM), Tetric N Flow Bulk Fill (TF), and Estelite Universal High Flow (EU)—and one conventional paste-like posterior composite (GC G-aenial Posterior; GP) were tested (*n* = 10 per subgroup). ISO specimens were evaluated for flexural strength (MPa) and MOD specimens evaluated for fracture load (N) using a three-point bending and fracture load test at 1 mm/min. Data were analyzed using one-way ANOVA and Tukey post hoc tests (α = 0.05).

**Results:**

Remarkably, the mechanical outcomes varied depending on the tested composite material and the specimen configuration (*p* < 0.05). In MOD shaped restorations, TF demonstrated the highest fracture load (1198.33 ± 188.83 N), followed by EU (1007.76 ± 182.37 N), GP (871.92 ± 227.67 N), and CM (776.10 ± 93.06 N). In ISO 4049 bar specimens, EU exhibited significantly higher flexural strength (85.84 ± 23.43 MPa) compared with CM (61.76 ± 2.99 MPa), TF (55.27 ± 12.04 MPa), and GP (54.19 ± 11.57 MPa) (*p* < 0.05). Material ranking differed between ISO bar and MOD specimen configurations.

**Conclusions:**

HFB resin composites showed flexural strength and fracture load values comparable to or higher than those of the conventional paste-like posterior composite. However, performance was material-dependent and influenced by specimen geometry. These findings highlight the importance of combining standardized and geometry-dependent testing approaches for a more comprehensive evaluation of restorative materials.

## Introduction

 The increasing patient demand for aesthetic and cost-effective restorations has led to a growing use of direct resin composite restorations [1]. Conventional resin composites are typically placed in 2 mm increments to reduce polymerization shrinkage, limit stress at the adhesive interface, and ensure an adequate depth of cure [2]. However, incremental placement requires multiple curing steps, which prolongs clinical time and increases technique sensitivity [3].

Bulk-fill resin composites were introduced to simplify restorative procedures and reduce treatment time while maintaining sufficient mechanical and esthetic properties. Due to increased translucency and optimized photo initiator systems, these materials can be applied in increments of up to 4–5 mm [4, 5], reducing layering steps and the risk of void formation. They have also been reported to exhibit a higher degree of conversion and lower polymerization shrinkage stress compared with conventional composites, attributed to modifications in the resin matrix and filler technology [6]. Their acceptable surface hardness and wear resistance support their use in posterior restorations and laboratory-based mechanical testing. Despite these favorable properties, further material developments were required to overcome the mechanical limitations associated with earlier formulations [7–9].

In this context, flowable bulk-fill composites were developed to improve handling and cavity adaptation. However, due to their relatively lower filler content, early flowable formulations often exhibited inferior mechanical properties and were recommended to be used in combination with a capping layer of conventional composite [4, 10, 11].

To overcome these limitations, high flowable bulk-fill (HFB) composites were developed to improve cavity adaptation and handling while maintaining sufficient mechanical performance and depth of cure. Unlike first-generation flowable composites, which often require coverage with a stronger restorative layer due to limited strength, HFB materials incorporate advanced resin formulations and filler technologies that enhance flexural and compressive properties to levels comparable with conventional paste-like composites [12–15], (https://www.kuraraynoritake.eu/lv/chairside). Therefore, these materials may be suitable for use in stress-bearing posterior regions, including occlusal surfaces.

Previous studies have demonstrated that highly filled flowable composites demonstrate acceptable mechanical performance when used as a single restorative material in posterior cavities [16–19]. However, evidence remains limited regarding the mechanical reliability of HFB composites, particularly when used alone and evaluated under clinically relevant cavity designs [20]. This highlights the need for further investigation using test methods that reflect both standardized material properties and clinically representative conditions.

Flexural strength is commonly assessed using the three-point bending test recommended by ISO 4049, which enables standardized comparison of resin-based composite materials independent of restorative cavity configuration [21–24]. While ISO bar specimens provide valuable information about intrinsic material properties, they do not fully reproduce the complex biomechanical environment of intraoral restorations, where cavity design and remaining tooth structure strongly influence stress distribution and failure patterns. In this context, mesio-occluso-distal (MOD) cavities represent a clinically challenging situation due to the extensive loss of tooth structure and reduced cuspal support, resulting in increased susceptibility to fracture under occlusal loading [25]. Therefore, evaluating both flexural strength in standardized ISO bar specimens and fracture load in cavity-shaped configurations provides complementary information about the intrinsic mechanical properties of the material and its structural behavior under clinically relevant loading conditions.

Accordingly, this study aimed to compare the flexural strength and fracture load of high-flow bulk-fill composites with a conventional paste-like posterior composite using standardized ISO 4049 bar specimens and MOD cavity-shaped models.

The null hypothesis tested in this study was as follows:

- H0: Composite material type would have no significant effect on flexural strength or fracture load.

## Materials and methods

### Study design and sample size calculation

This laboratory study assessed both intrinsic strength and fracture resistance of one conventional composite as control; GC G-aenial Posterior (GP) and three high viscosity bulkfill composite; Clearfil Majesty Es High Flow (CM), Tetric N Flow Bulk Fill (TF), Tokuyama Estelite Universal High Flow (EU) restorations. Independent variable was restorative material. Dependent variables were flexural strength and fracture load values determined by a universal testing machine.

Sample size was determined by an a priori power analysis using G*Power software (version 3.1.9.7; Heinrich Heine University, Düsseldorf, Germany). The analysis was based on an F test for one-way ANOVA, assuming an effect size of f = 0.405, a significance level of α = 0.05, and a statistical power of 1 − β = 0.85. The minimum required sample size was calculated as 40 specimens for each test.

### Specimen preparation

The composition and application protocols of the tested materials are provided in Table 1.

**Table 1:** The composition of the materials and application methods

Type of Composites	Material	Groups	Shade	Lot Number	Composition	Filler content (% by weight)	Curing time	Manufacturer
	Clearfil Majesty Es High Flow	CM	A2	920,014	TEGDMA, hydrophobic aromaticdimethacrylate, silanated barium glass filler, pre-polymerized organic filler	78 wt%	20 s	Kuraray Noritake Dental, Tokyo,Japan
High Flow Bulk Fill Composites	Tetric N Flow Bulk Fill	TF	A2	Z070CG	Bis-GMA, UDMA, DMA Inorganic filler: barium glass, ytterbium fluoride, silica (particle size 0.04–3 μm)	57.5 wt%	20 s	Ivoclar AG; Schaan, Liechtenstein
	Estelite Universal High Flow	EU	A2	151E94	Bis-GMA, Bis-MPEPP, TEGDMA, UDMA, 200 nm spherical SiO2*ZrO2	69 wt%	10 s	Tokuyama Dental, Tokyo, Japan
Conventional Paste-like Composites	G-aenial Posterior	GP	A2	2,307,051	Bis-EMA, UDMA, dimethacrylate	77% wt	20 s	GC Corporation, Tokyo, Japan

*TEGDMA *Triethylene Glycol Dimethacrylate, *Bis-GMA *Bisphenol A-Glycidyl Methacrylate, *UDMA *Urethane Dimethacrylate, *D3MA *Decamethacrylate, *Bis-MPEPP *Bisphenol A Polyethylene Glycol Ether Dimethacrylate, *Bis-EMA *Ethoxylated Bisphenol A Dimethacrylate

Specimens were divided into two experimental configurations (*N* = 40 each): MOD shaped restorations and ISO 4049 standard bar-shaped specimens. Within each configuration, specimens were randomly allocated to four restorative material groups (*n* = 10): Clearfil Majesty Es High Flow (CM), Tetric N Flow Bulk Fill (TF), Tokuyama Estelite Universal High Flow (EU), and GC G-aenial Posterior (GP), which served as the control group.

**Fig. 1:**
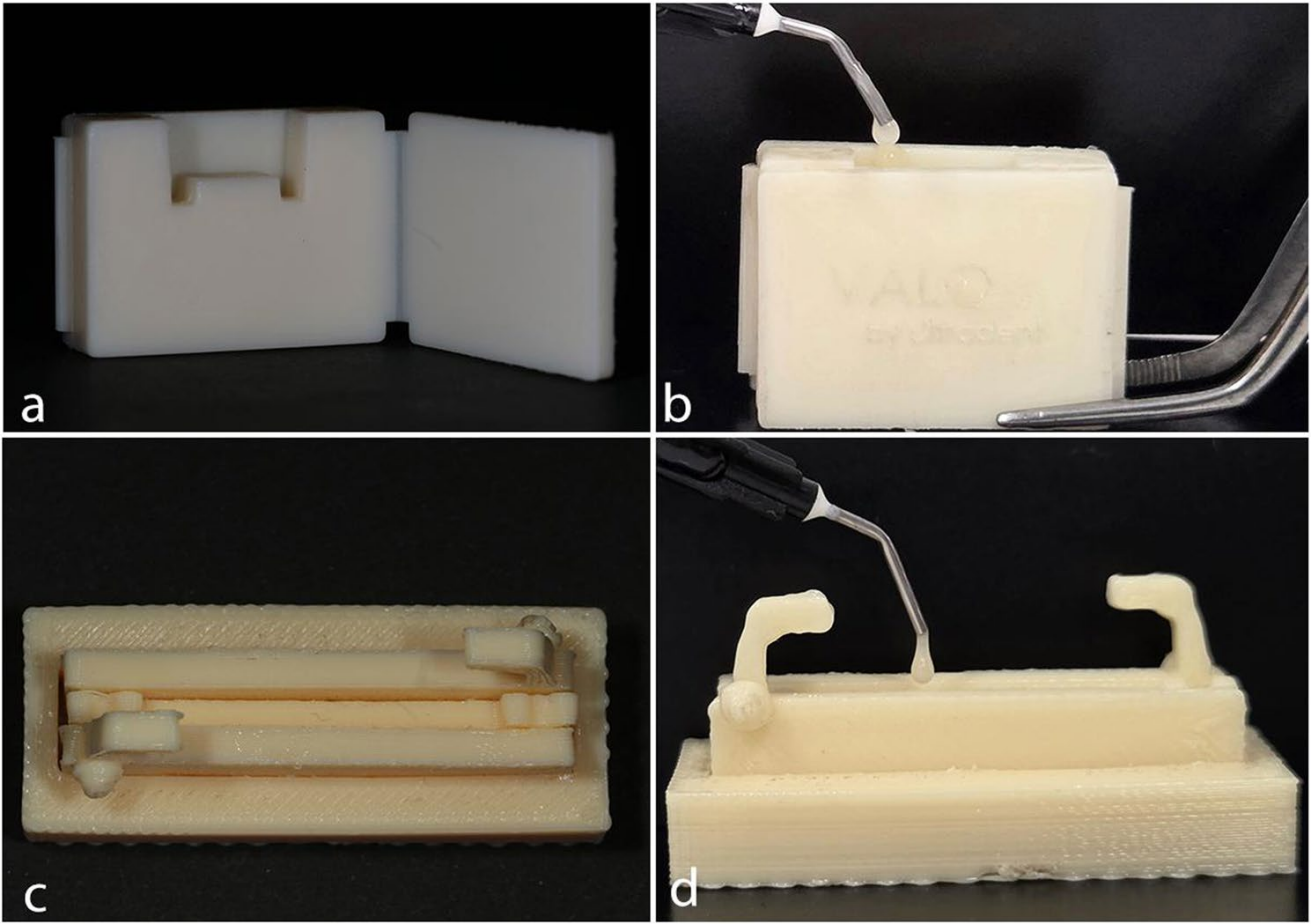
Prefabricated MOD shaped mold (**a**,** b**) (Ultradent, South Jordan, UT, USA) and 3D-printed ISO 4049 standard bar mold (**c**,** d**) used for specimen preparation

MOD shaped restorations (*n* = 40) were prepared using prefabricated molds (Ultradent, South Jordan, UT, USA) (Fig. 1a–b). These specimens consisted of composite-only structures fabricated in standardized molds rather than restorations placed in extracted teeth. The mold dimensions were 12 mm (length) × 7 mm (depth) × 5 mm (width). In the group, bulk-fill composites were placed in two increments to fill the entire cavity depth. The first increment (4 mm thickness) was applied and light-cured, followed by a second increment (3 mm thickness) which was subsequently light-cured.

Polymerization was performed using an LED light-curing unit (Valo, Ultradent Products Inc., South Jordan, UT, USA) in standard mode, with an output intensity of 850–1000 mW/cm² and a spectral range of 395–480 nm. Light-curing times were applied according to the manufacturers’ instructions for each material (Table 1). The irradiance output was verified before each use with a radiometer (Hilux Ledmax curing meter, Benlioğlu Dental, Ankara, Turkey). Each specimen was light-cured from both mesial and distal directions in two separate exposures. A Mylar strip and a 1-mm-thick glass plate were placed over the mold to standardize the distance between the light-curing tip and the composite surface.

After specimen preparation, all MOD specimens were stored in distilled water at 37 °C for 24 h prior to mechanical testing.

ISO 4049 standard bar specimens (*n* = 40) were fabricated using custom 3D-printed molds produced with a Formlabs 3D printer (Formlabs, Somerville, MA, USA) (Fig. 1c–d). Bar dimensions were 25 mm (length) × 2 mm (width) × 2 mm (height). Composite material was placed into the mold in a single increment. Due to the 8-mm diameter of the light-curing tip, polymerization was performed in three consecutive overlapping exposures with slight overlap between adjacent irradiations to ensure complete coverage of the entire specimen surface. Light curing was carried out using the same LED curing unit, following the manufacturers’ instructions (Table 1). All specimens were stored in distilled water at 37 °C for 24 h before testing.

### Fracture load test

**Fig. 2:**
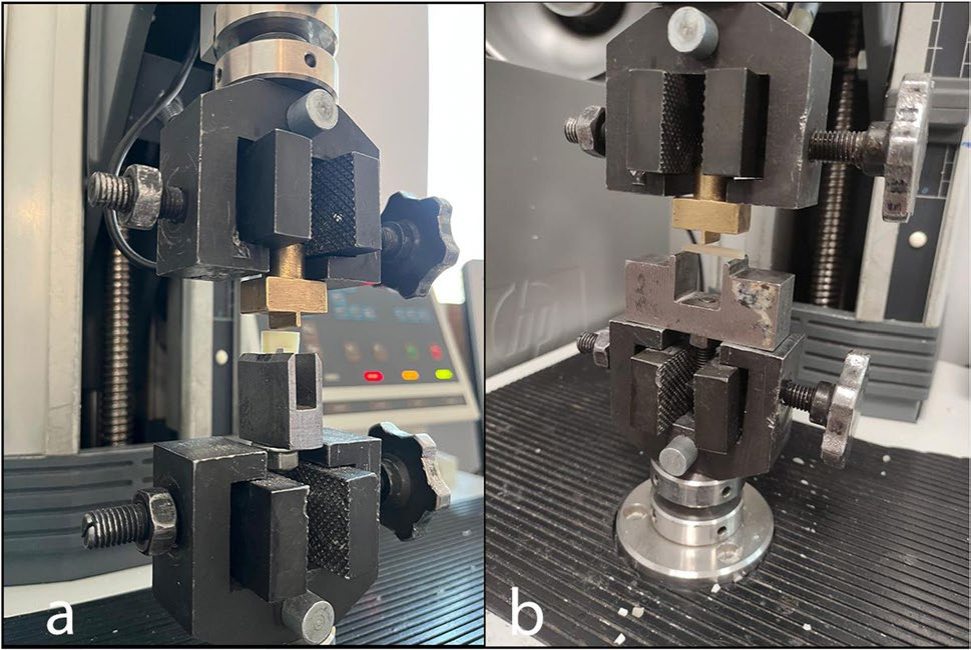
**a** Lloyd LRX Universal Testing Machine (Lloyd Instruments, UK) used for flexural strength testing with a three-point bending setup: Testing configuration for ISO bar-type specimens. **b** Lloyd LRX Universal Testing Machine (Lloyd Instruments, UK) used for fracture load testing with a three-point bending setup: Testing configuration for MOD shaped restoration

**Fig. 3:**
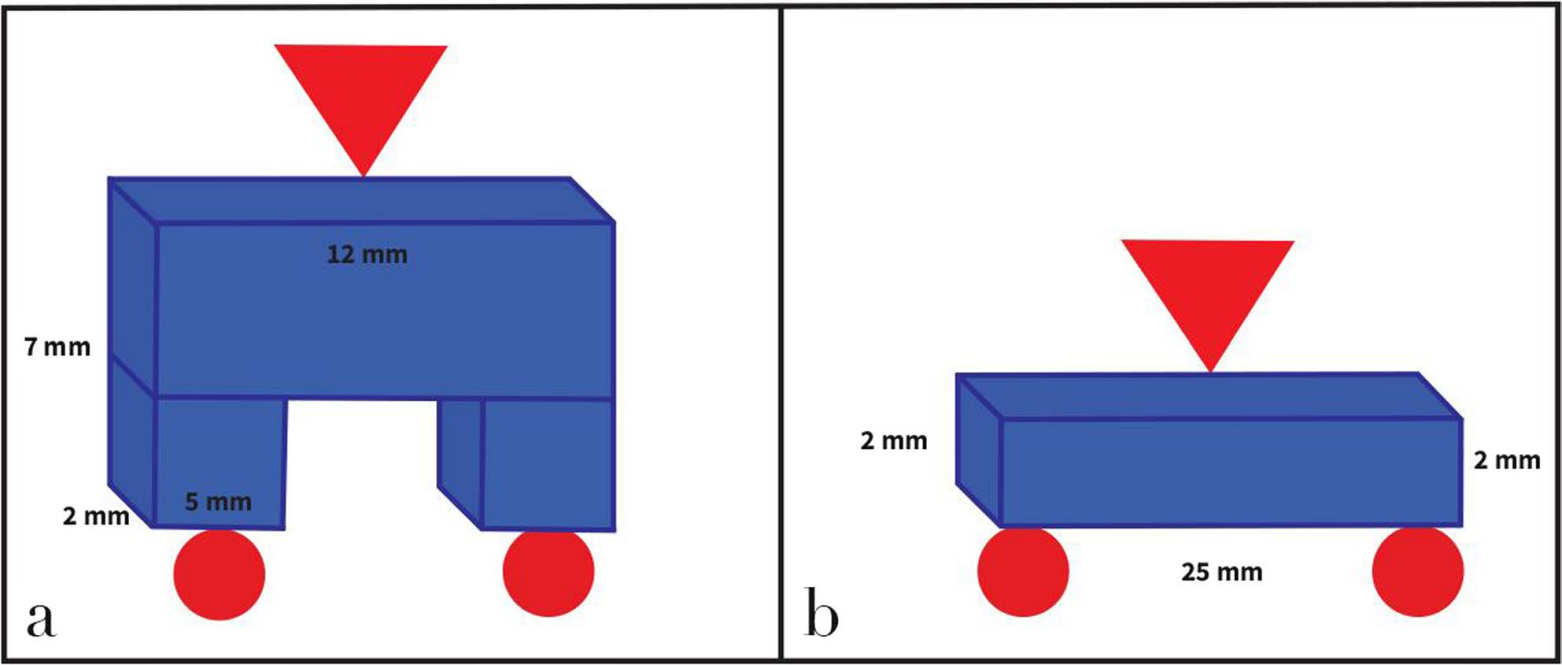
**a **Schematic illustration of the fracture load test setup: MOD cavity-shaped restoration (12 × 7 × 5 mm). **b** Schematic illustration of the three-point bending test setup: ISO 4049 standard bar-shaped specimen (25 × 2 × 2 mm)

Fracture load of the MOD shaped restorations was measured using the same universal testing machine (Lloyd LRX, Lloyd Instruments Ltd., Fareham, UK) under a three-point bending setup with a span length of 20 mm and a crosshead speed of 1 mm/min. The maximum load at failure was recorded in Newtons (N) (Figs. 2a and 3a).

### Three-point bending test

Mechanical testing was performed using a universal testing machine (Lloyd LRX, Lloyd Instruments Ltd., Fareham, UK) in a three-point bending setup with a span length of 20 mm. All specimens were tested at a crosshead speed of 1 mm/min at room temperature (23 ± 2 °C). This loading speed falls within the range recommended by ISO 4049 for flexural testing of resin-based restorative materials (0.75 ± 0.25 mm/min) [23, 26, 27].

For ISO bar specimens, flexural strength was calculated in megapascals (MPa) according to ISO 4049 using the following formula: σ = 3FL ​/2bh^2^ where σ is the flexural strength (MPa), F is the maximum load at fracture (N), L is the span length (mm), b is the specimen width (mm), and h is the specimen height (mm). The maximum load at fracture was recorded for each specimen and used to calculate flexural strength values (Figs. 2b and 3b).

### Statistical analysis

Statistical analysis was performed using IBM SPSS Statistics software (version 22.0; IBM Corp., Armonk, NY, USA). Normality of the data was evaluated using the Shapiro–Wilk test, and homogeneity of variance was assessed using Levene’s test.

Since the data showed normal distribution and homogeneity of variances, one-way analysis of variance (ANOVA) was used for statistical comparisons. For each specimen configuration, differences among restorative material groups were analyzed accordingly. When statistically significant differences were detected, Tukey’s post-hoc test was applied. The level of significance was set at *p* < 0.05.

## Results

**Fig. 4:**
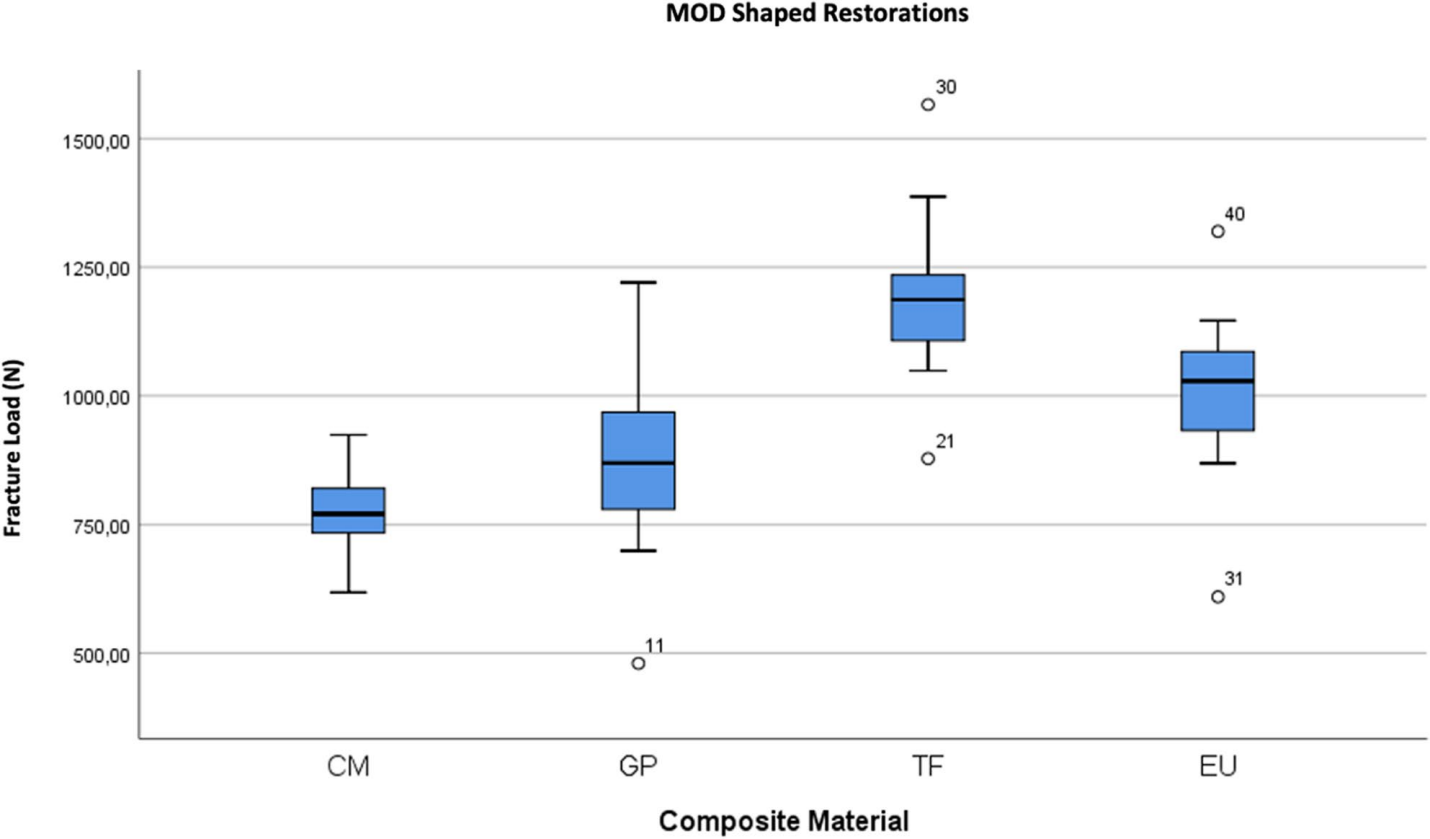
Fracture load (N) distribution of the tested composites in MOD shaped restorations

**Fig. 5:**
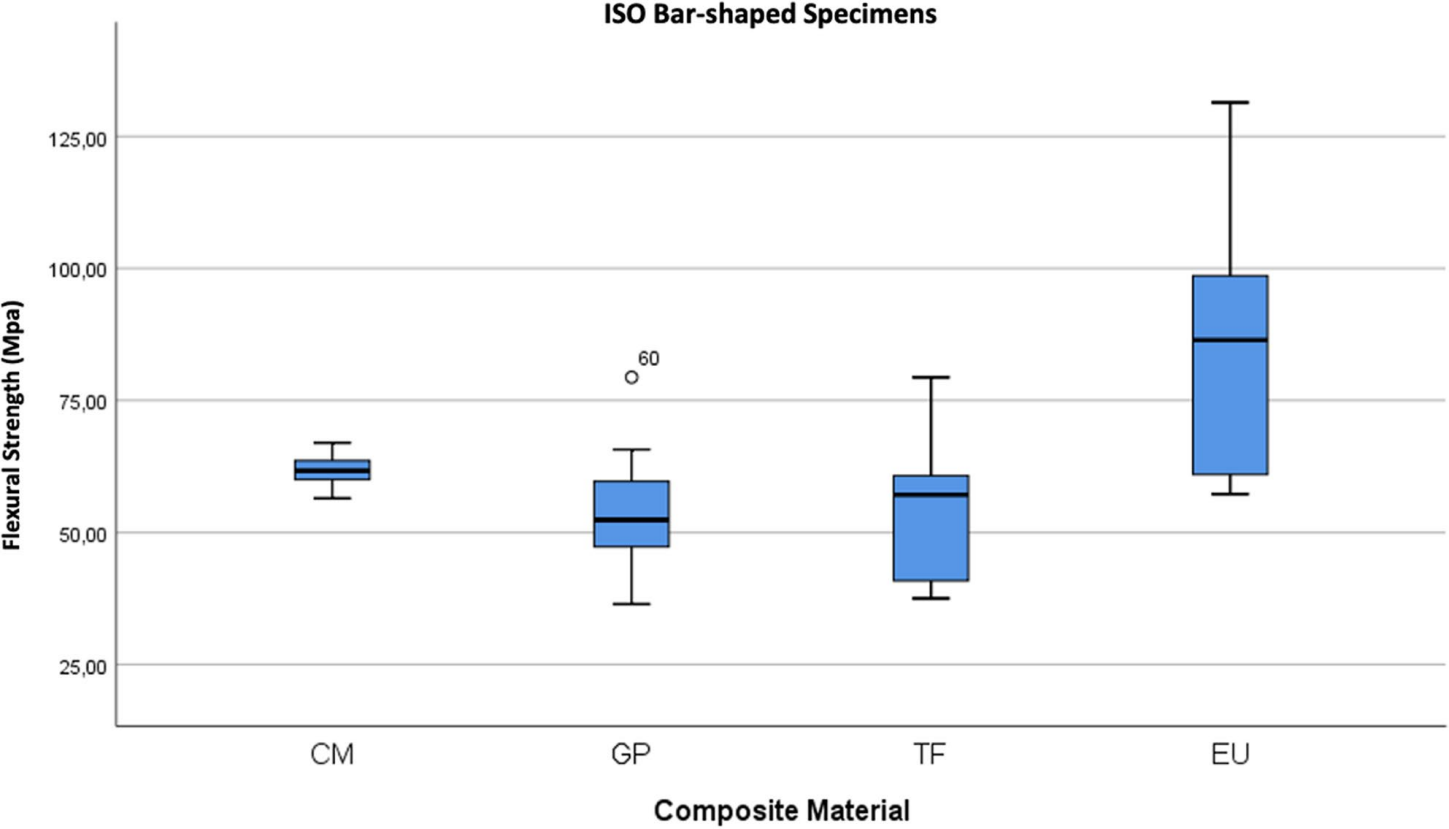
Flexural strength (MPa) distribution of the tested composites in ISO 4049 bar-shaped specimens

The results of the one-way ANOVA are presented in Tables 2 and 3. Statistically significant differences among the tested composite materials were detected for both the MOD shaped restorations and the ISO 4049 bar specimens (*p* < 0.05) (Figs. 4 and 5).

**Table 2:** Results of the one-way ANOVA for MOD cavity-shaped restorations

	MOD shaped restorations (N)						Anova		
	*n*	Mean	Median	Minimum	Maximum	ss	F	*p*	Tukey
CM	10	776.1	776.3	617.9	924.7	93.06	10,36	< 0,001	2-3 4-1 3-1
GP	10	871.92	866.70	480.24	1220.19	227.67			
TF	10	1198.33	1167.83	878.40	1566.16	188.83			
EU	10	1007.76	1038.26	609.54	1319.66	182.37			

**Table 3:** Results of the one-way ANOVA for ISO 4049 bar-shaped specimens

	**ISO bar specimens (MPa)**						**Anova**		
	***n***	**Mean**	**Median**	**Minimum**	**Maximum**	**ss**	**F**	***p***	**Tukey**
CM	10	61.76	61.62	56.51	67.03	2.993	10,43	<0,001	2-4 4-3 4-1
GP	10	54.19	53.81	36.47	79.38	11.57			
TF	10	55.27	52.72	37.54	79.35	12.04			
EU	10	85.84	80.77	57.31	131.43	23.43			

One-way ANOVA revealed statistically significant differences in fracture load among the tested composite materials in the MOD shaped restorations (*p* < 0.001) (Table 2). The TF group exhibited the highest fracture load values, followed by the EU, GP, and CM groups. Pairwise comparisons showed that the CM group had significantly lower fracture load values than the TF and EU groups (*p* < 0.05). In addition, the GP (control) group demonstrated significantly lower fracture load values than the TF group (*p* < 0.05). No other significant differences were observed among the remaining groups (*p* > 0.05).

One-way ANOVA revealed statistically significant differences in flexural strength among the tested composite materials in the ISO 4049 bar specimens (*p* < 0.001) (Table 3). The EU group exhibited the highest flexural strength values, followed by the CM, TF, and GP groups. Pairwise comparisons indicated that the EU group showed significantly higher flexural strength values than the CM, TF, and GP groups (*p* < 0.05). No significant differences were observed among the remaining groups (*p* > 0.05).

## Discussion

The present study evaluated and compared the flexural strength and fracture load of high-flow bulk-fill (HFB) composites and a conventional paste-like composite using two specimen configurations: standardized ISO 4049 bar specimens and MOD shaped restorations. The results demonstrated that the tested composite materials showed significantly different flexural strength and fracture load values; therefore, the null hypothesis was rejected.

Bulk-fill composites enable simplified restorative procedures through increased depth of cure and reduced application time, which supports their use in posterior restorations [20]. In the present study, these materials were evaluated using two different specimen configurations: standardized ISO 4049 bar specimens and MOD shaped restorations, in order to assess their mechanical behavior under both standardized and geometry-dependent conditions.

High-flow bulk-fill composites are used due to their low viscosity, which enhances adaptation to cavity walls and reduces the risk of void formation. These materials allow for uniform adaptation and improved contact with the dentin surface, particularly in wide MOD-type cavities or areas with limited access [28]. Enhanced flowability contributes to a more uniform redistribution of polymerization shrinkage stress during polymerization, which in turn mitigates stress concentration at the adhesive interface. In line with this mechanism, previous studies have demonstrated that improved flow and homogeneous composite distribution promote more uniform stress distribution and may further reduce shrinkage-induced stress development [29, 30]. Moreover, their high translucency permits sufficient light transmission, ensuring adequate curing even in deeper regions. From a clinical perspective, the use of high-flow bulk-fill composites shortens application time and supports long-term restoration success by minimizing marginal leakage and microleakage [31]. These properties make high-flow bulk-fill composites suitable for evaluating mechanical behavior under different specimen configurations.

In the MOD shaped restorations, significant differences in fracture load were observed among the tested materials. The TF group exhibited the highest fracture load values, followed by the EU and GP groups, whereas the CM group showed the lowest mean values. These findings suggest that material composition plays a critical role in mechanical performance under geometry-dependent loading conditions. However, this trend was not consistent across all materials. The comparable performance of the control group (GP) indicates that factors beyond filler content such as filler morphology, particle–matrix interaction, and polymer network formation may play a decisive role in determining fracture load.

In the ISO 4049 bar specimens, the EU group demonstrated significantly higher flexural strength than the other materials, whereas no significant differences were observed among the remaining groups. The superior performance of EU under standardized conditions suggests higher intrinsic mechanical strength; however, its relative performance differed under geometry-dependent conditions. These findings indicate that different testing configurations provide complementary information: ISO bar testing primarily evaluates intrinsic material properties under uniform stress distribution, whereas MOD shaped restorations reflect mechanical behavior under geometry-dependent loading conditions.

The mechanical behavior of HFB composites is influenced not only by overall filler content but also by filler morphology and the quality of filler–matrix interaction. Variations in particle size, filler shape, and filler–matrix interaction affect stress transfer within the composite and influence its mechanical performance [22]. Consequently, HFB composites with comparable filler loading may exhibit distinct mechanical behavior depending on their microstructural characteristics, which may partially explain the differences observed in the present study.

The superior flexural strength of the EU group under ISO bar testing may be attributed to its 200-nm spherical SiO₂·ZrO₂ supra-nano filler technology. Spherical fillers allow more homogeneous packing within the resin matrix and distribute stresses more uniformly than irregular-shaped glass fillers, minimizing the stress-concentration points and crack-initiation sites that develop at sharp filler edges [22, 32]. The inclusion of zirconium dioxide additionally enhances filler hardness, which may further contribute to the material’s mechanical stability under uniform loading conditions [33]. The highest fracture load observed in the TF group under the MOD configuration may be explained by the Bis-GMA/UDMA balance within its resin matrix. Bis-GMA provides cross-linking density and stiffness, whereas UDMA contributes to flexibility, a higher degree of conversion, and greater fracture toughness through hydrogen bonding [34]. This combination yields a polymer network capable of tolerating greater deformation before failure, an advantageous property under the geometry-dependent loading of the MOD configuration [22].

In the ISO 4049 bar specimens, the flexural strength values of the EU group exceeded 80 MPa, which is generally regarded as the minimum flexural strength threshold defined by ISO 4049 for posterior restorative materials [35]. Notably, the remaining materials fell below this threshold under ISO bar testing, although they still demonstrated adequate fracture load values under MOD-shaped configuration further supporting the geometry-dependence of mechanical outcomes.

The discrepancy between ISO and MOD results indicates that materials performing well under standardized testing conditions may not exhibit equivalent behavior in geometry-dependent configurations. Similar findings have been reported by Chai et al. [13], who demonstrated that specimen geometry and stress distribution significantly influence fracture patterns in composite materials. Therefore, evaluating both standardized and geometry-dependent models provides a more comprehensive understanding of restorative material behavior. The existing literature has reported considerable variability in strength testing results of dental resin-based composites, primarily attributable to differences in testing methodologies and specimen geometries. This variability supports the use of multiple testing configurations, as applied in the present study, to obtain complementary information on fracture behavior [36].

When comparing HFB composites with the conventional paste-like composite (GP), certain HFB materials—particularly TF and EU demonstrated comparable or superior mechanical performance. These findings support recent literature indicating that advanced high-flow formulations can now match traditional hybrid composites in terms of mechanical reliability [37, 38]. Earlier generations of flowable composites were primarily indicated as liners or base materials due to their limited mechanical properties [39, 40]; however, recent technological advancements, such as nano-filler incorporation and optimized photo initiator systems, have significantly enhanced their flexural and compressive strength [33].

In contrast to the present findings, some studies using MOD-restored molars have reported no significant differences in mechanical performance between bulk-fill composites of varying viscosities and conventional composites. Such discrepancies are likely related to differences in experimental design and testing protocols, highlighting the sensitivity of mechanical outcomes to test configuration [41].

Although numerous studies have investigated the flexural strength of bulk-fill resin composites, the majority have focused on conventional bulk-fill formulations using standardized bar-shaped specimens. In contrast, evidence regarding the mechanical performance of HFB composites remains limited, particularly when these materials are evaluated under different specimen geometries. Moreover, studies simultaneously assessing HFB composites using both standardized ISO bar specimens and geometry-dependent configurations are scarce. This lack of comprehensive data highlights the need for experimental approaches that integrate multiple testing geometries to better elucidate the mechanical behavior of HFB materials.

Consequently, the present results are consistent with previous reports suggesting that modern HFB composites may exhibit mechanical performance comparable to conventional materials under the tested conditions, provided that adequate material thickness and proper curing protocols are maintained [20, 31, 42].

From a practical standpoint, these two testing approaches provide complementary, rather than interchangeable, information. The ISO 4049 bar specimen test is designed to evaluate intrinsic material properties under standardized and reproducible conditions, making it particularly useful for material development, quality control, and inter-study comparisons. In contrast, the MOD-shaped configuration, while still in vitro, introduces geometry-dependent stress distribution patterns that more closely resemble the loading conditions encountered in clinical MOD restorations. Therefore, geometry-based testing may offer incremental value when interpreting material behavior in the context of clinically challenging cavity designs. However, as neither configuration incorporates adhesive interfaces or a dentin substrate, the findings should be interpreted as material-level observations rather than direct predictors of clinical performance.

This study is limited by its in vitro design, which cannot fully reproduce the complex oral environment, including thermal fluctuations, masticatory loading, and occlusal wear. Although both ISO bar and MOD shaped restorations configurations were employed to represent standardized and geometry-dependent conditions, the MOD specimens consisted of composite-only structures without dentin support or adhesive interfaces. Therefore, the findings cannot be directly extrapolated to in vivo performance. Future studies should also evaluate the long-term mechanical stability of high-flow bulk-fill composites under thermomechanical aging and cyclic loading on permanent teeth. In addition, assessment of adhesive interface integrity and marginal sealing ability would further enhance understanding of their functional durability.

## Conclusions

Within the limitations of this in vitro study, high-flow bulk-fill composites demonstrated mechanical performance comparable to or higher than that of the conventional paste-like posterior composite. However, significant differences were observed among the tested high-flow bulk-fill materials, and performance varied depending on specimen configuration, with distinct outcomes in ISO 4049 bar specimens and MOD shaped restorations. These findings suggest that both material formulation and testing geometry influence mechanical behavior. Further studies incorporating thermomechanical aging, cyclic loading, and additional specimen configurations are required to better predict the long-term clinical performance of high-flow bulk-fill composites. 

## Data Availability

The data supporting the findings of this study are available from the corresponding author upon reasonable request.

## Abbreviations

HFB: High-flow bulk-fill
ISO: International Organization for Standardization
CM: Clearfil Majesty Es High Flow
TF: Tetric N Flow Bulk-fill
EU: Estelite Universal High Flow
GP: G-aenial Posterior
